# Risk factors for bacterial catheter colonization in regional anaesthesia

**DOI:** 10.1186/1471-2253-5-1

**Published:** 2005-03-17

**Authors:** Astrid M Morin, Klaus M Kerwat, Martina Klotz, Roswitha Niestolik, Veronika E Ruf, Hinnerk Wulf, Stefan Zimmermann, Leopold HJ Eberhart

**Affiliations:** 1 Department of Anaesthesiology and Critical Care Medicine, Philipps University, Baldingerstrasse 1, D-35043 Marburg, Germany; 2 Institute of Medical Microbiology and Hygiene, Philipps-University, Baldingerstrasse 1, D-35043 Marburg, Germany

## Abstract

**Background:**

Although several potential risk factors have been discussed, risk factors associated with bacterial colonization or even infection of catheters used for regional anaesthesia are not very well investigated.

**Methods:**

In this prospective observational trial, 198 catheters at several anatomical sites where placed using a standardized technique. The site of insertion was then monitored daily for signs of infection (secretion at the insertion site, redness, swelling, or local pain). The catheters were removed when clinically indicated (no or moderate postoperative pain) or when signs of potential infection occurred. After sterile removal they were prospectively analyzed for colonization, defined as > 15 colony forming units.

**Results:**

33 (16.7%) of all catheters were colonized, and 18 (9.1%) of these with additional signs of local inflammation. Two of these patients required antibiotic treatment due to superficial infections. Stepwise logistic regression analysis was used to identify factors associated with catheter colonization. Out of 26 potential factors, three came out as statistically significant. Catheter placement in the groin (odds-ratio and 95%-confidence interval: 3.4; 1.5–7.8), and repeated changing of the catheter dressing (odds-ratio: 2.1; 1.4–3.3 per removal) increased the risk for colonization, whereas systemic antibiotics administered postoperatively decreased it (odds ratio: 0.41; 0.12–1.0).

**Conclusion:**

Colonization of peripheral and epidural nerve catheter can only in part be predicted at the time of catheter insertion since two out of three relevant variables that significantly influence the risk can only be recorded postoperatively. Catheter localisation in the groin, removal of the dressing and omission of postoperative antibiotics were associated with, but were not necessarily causal for bacterial colonization. These factors might help to identify patients who are at increased risk for catheter colonization.

## Background

Questions about the infection control practices of anaesthesiologists are as old as our specialty and raised as early as 1873 by Skinner [1]. To control infectious complications associated with regional anaesthesia, current recommendations are based on national organizations. Although several risk factors have been discussed, risk factors associated with bacterial colonization or even infection that could guide such recommendations have not been investigated systematically so far or clinical trials had too few patients to draw meaningful conclusions. Among the risk factors that have been suspected to abet catheter infection are age, pre-existing diseases (e.g. diabetes mellitus, drug abuse, alcoholism), sepsis, and medical treatment compromising the immune response [2-4], site of catheter insertion [2,3,5], technically difficult catheter insertion with development of an asymptomatic haematoma that may later become the focus of bacterial colonization [6], filter changing manoeuvres or disconnecting the system [7] and duration of catheter use[5]. Prophylactic antibiotics, use of local anaesthetic solution with bacteriostatic effect and antimicrobial filters are thought to decrease the risk of infection [8,9].

Thus, the purpose of this observational study was to prospectively determine the incidence of catheter bacterial colonization and infectious complications in postoperative patients having peripheral nerve or epidural catheters at different sites, and to identify factors associated with bacterial colonization of peripheral or epidural nerve catheters.

## Methods

This prospective study was approved by the local ethics committee and informed consent was obtained from each patient. Consecutive patients scheduled for elective surgery (orthopaedic, cardiac, visceral and urologic surgery) receiving various peripheral or epidural catheters were enrolled in this study over a period of 5 months. All catheters were placed preoperatively in the operating room or in the pre-anaesthetic holding area. No patients for chronic pain therapy were considered.

### Catheter insertion

The procedure for catheter insertion was standardized and carried out with a standardized aseptic technique, according to the guidelines of the German Robert-Koch-Institution. In short these included wearing a surgical hood, face mask, sterile gloves after hand disinfection, a sterile coat, and using a large sterile drape covering the insertion site. The skin was disinfected for at least one minute by wiping or by spraying (at the anaesthetist's discretion) with Cutisept^® ^(contains in 100 g: 2-Propanol 63 g, benzalkoniumchlorid 0,025 g, cleaned water and dyestuff). This disinfectant is suitable for all sites and recommended by the DGHM (Deutsche Gesellschaft für Hygiene und Mikrobiologie = German Society for Hygiene and Microbiology).

Bacterial filters provided with the sets were attached to all catheters in a sterile manner. The catheter insertion sites were covered with a sterile transparent dressing that permits the escape of moisture from beneath the dressing (Tegaderm^®^, consisting of polyurethan). In case of blood sequestration on the insertion site, sterile gauze was placed under the dressing. No antimicrobial prophylaxis was administered specifically for the nerve catheter insertion, but nearly all patients received a single-shot perioperative antibiotic prophylaxis after catheter placement before surgery. In orthopaedic and cardiac surgery, cefuroxim 1.5 g, and in visceral and urologic surgery a fix combination of 2 g ampicillin + 1 g sulbactam was administered intravenously.

### Perioperative catheter management

An initial bolus dose of a local anaesthetic was injected preoperatively. Patients with a peripheral regional catheter received a mixture of 20 ml prilocaine 1% and 20 ml ropivacaine 0.75%, and patients with an epidural catheter had 10 ml of ropivacaine 0.5–0.75% after an initial test dose of 2–3 ml bupivacaine 0.5%. Then a continuous infusion of ropivacaine 0.2% (5–15 ml/h for peripheral regional anaesthesia and 4–10 ml/h for epidural anaesthesia) was started in the postanaesthesia holding area and continued on the ward.

The catheter management postoperatively was standardized and carried out by the acute pain service by one of the authors (A.M.M.). The catheters were kept in place as long as clinically indicated, depending on a daily evaluation of the intensity of pain (aiming at a pain level of 3 cm or less on a 10 cm visual analogue scale) and the evaluation of the insertion site. For these purposes the patients were visited twice a day and the dressing was inspected and palpated. The dressings were changed only if necessary. This was defined as follows: first the site of catheter insertion was contaminated with blood, second there was a wet chamber under the dressing, or third the dressing was about to peel away. The algorithm of care used after unintentional dressing removal as well as for intended replacements was disinfection of the skin by spraying on the insertion site with Cutisept^®^, cleaning the insertion site with sterile compresses and let dry for at least one minute, then fixing a new dressing. If a catheter was obviously disconnected for a short time (less than 30 minutes), it was cleaned and disinfected about 10 cm distant from the catheter end, cut with sterile scissors and reconnected using a new sterile connector and a new bacterial filter. If the time period since disconnection was unclear, the catheter was removed. Otherwise no filter change was performed, even if the catheter was in place for a longer time.

Measurements of body temperature and a neurological examination were performed at least once a day as long as the catheter was in situ and again two days after its removal.

### Catheter removal

The catheters were removed under aseptic conditions. To prevent bacterial contamination of catheter tips, the skin was disinfected with Cutisept^® ^for one minute. Only when the skin had dried completely the catheter was removed to avoid direct contact of the catheter tip with the disinfectant agent. The distal catheter tip was cut with sterile scissors, placed in a sterile transport medium and transferred immediately to the microbiology laboratory.

### Bacteriological methodology

Semi-quantitative culture techniques were used as described by Maki et al. [10]. The catheter segment was rolled several times across the surface of an agar plate and incubated overnight at 35°C under aerobic conditions. Then, the same catheter segment was immersed in 5 ml thioglycolate broth. After overnight incubation at 35°C aliquots of the broth were transferred to a 5% sheep blood agar plate and a MacConcey agar plate (Becton Dickinson, Heidelberg, Germany) and again incubated at 35°C for 24 h. Colony forming units (CFU) were counted and identified by standard microbiological methods. The presence of more than 15 CFU of a single organism per catheter was considered colonization, and if accompanied by signs of local inflammation (redness, swelling, and pain with pressure or tapping on the insertion site) it was defined as local infection.

### Collection and processing of the data

To allow comprehensive analysis of potential factors associated with bacterial colonization, a large amount of clinical variables were recorded prospectively. These are listed in table 1. Some of them were pre-processed to reduce the load for the multifactorial statistical analysis. E.g., the patients' weight and height were used to calculate the body-mass-index (BMI). Furthermore, factors that were observed with a low incidence and therefore having no realistic chance to provide statistical significance in the univariate and in the multivariate analysis (history of infectious disease of the skin (n = 8) and infection with other catheter material in the past (n = 3) were analyzed separately and after merging them into an additional dummy variable. The same strategy was used for factors known to provoke surgical wound infection [11]. These were diabetes mellitus (n = 24), chronic steroid medication (n = 9), and cancerous disease (n = 49) [12]. The anatomical site of catheter insertion was grouped using the incidences of catheter colonization in a descriptive univariate analysis. Several attempts to group the different catheter techniques were used but finally the best discriminating power was achieved by summarizing catheters located in the groin (femoral nerve catheters and sciatic nerve catheters inserted by the anterior approach described by Meier et al[13]) against all other techniques.

**Table 1:** Results of the stepwise logistic regression analysis, where all potential risk factors are included during the first step and subsequently removed if not significant (p > 0.05). *Please note that p-values at removal of the parameter must not necessarily have the same order than the step at which the factor was removed, since the logistic model recalculates at each step.

Clinical parameter		Removed at step	Removed with an odds-ratio of	Removed with a p-value of
Sex (female vs. male gender)		2	0.89	0.88
Age (per decade)		12	0.84	0.34
Body mass index (per unit kg·m^-2^)		15	0.95	0.57
ASA-status III and IV versus I and II		18	1.48	0.22
Diabetes mellitus		7	3.72	0.99
Cancerous disease		19	0.48	0.18
Chronic systemic corticosteroid medication		20	1.89	0.13
	"immune suppression" (any of the three afore mentioned diseases)	5	1.35	0.99
Skin abscess in the past		3	0.74	0.87
Infection with other catheter material in the past		16	0.17	0.26
Easy perspiration^1^		11	2.33	0.32
	Any of the three afore mentioned factors	14	1.64	0.43
Localization of the catheter ("clean" vs. "dirty")^2^		8	0.38	0.37
Puncture site in the groin		Not removed	3.39	0.004
Epidural catheter vs. peripheral nerve catheter		17	1.24	0.11
Disinfection technique (wiping versus spraying)		9	1.88	0.50
Number of attempts during catheter placement		10	1.49	0.38
Fixation technique (tunneling versus suturing)		1	0.97	0.97
Intraoperative corticosteroid medication		4	1.40	0.75
Intraoperative single shot antibiotic administration		6	2.34	0.47
Postoperative antibiotic therapy (at least 3 days or until removal of the catheter)		Not removed	0.41	0.05
	Perioperative antibiotic therapy (intra- & postoperative)	23	0.43	0.09
Duration of catheter use (per day)		21	1.17	0.16
Accidental disconnection of the catheter		22	0.78	0.19
Accidental removal of the catheter dressing (n =)		15	0.50	0.37
Intentioned replacements of the catheter dressing (n =)		13	0.83	0.31
	Removal of the catheter dressing (intentionally or unintentionally; n =)	Not removed	2.12	0.001

1) Easy perspiration (e.g. getting bathed in sweat every night, sweating without moving very much) with the danger of easy removal of the dressing

2) Localization of the catheter ("dirty" = groin, axilla, interscalene, epidural below Th3; "clean" = epidural above Th3, paravertebral, psoas compartment, posterior and distal sciatic nerve, infraclavicular) based on the distribution of sebaceous glands on the skin [21]

### Statistical analysis

Twenty six potentially relevant variables were entered into a stepwise backward logistic regression analysis using the maximum likelihood method. They are listed in table 1 with the order of removal from the model and the odds-ration and p-value, respectively. The goodness of fit of the regression model was judged using Nagelkerkes's R^2^. All analyses were performed using JMP 5.1 for Windows (SAS Institute Inc., Cary, NC, USA) and SPSS 11.5 for Windows (SPSS Inc., Chicago, Ill, USA).

## Results

### Demographic data

A total of 200 catheters from 191 patients were initially enrolled in the study. Two catheters were excluded because they were not removed in accordance with the aseptic technique. Thus, 198 catheters from 189 patients could be analyzed. Five patients for total knee replacement had double catheterization (sciatic and femoral nerve catheter) and four patients had a repetitive intervention within a couple of months with the same technique.

Catheters were removed between day 0 (if a planned extensive surgery was modified intraoperatively into a smaller one not requiring postoperative analgesia via a catheter), and day 31 after an invasive procedure. In mean, catheters were in use for 3.7 days (standard deviation: 3.0). The median and the 25^th ^/ 75^th ^percentile were: 3; 2 / 5. This time period was not different in colonized catheters (mean: 3.8 ± 2.1 days) and uncolonized catheters (mean: 3.7 ± 3.1 days).

### Bacteriological results

Of 198 catheters analysed, 47 (23.7%; 95%-confidence interval: 18–30%) were not sterile. A heterogeneous flora of bacteria could be detected. In most cases (78.7%) these were normal non pathogenic skin flora. Coagulase negative staphylococci were most often detected, and only 21.3% were optional pathogenic microorganisms (table 2). In 33 patients (16.7%; 95%-confidence interval: 12–23%) there were more than 15 CFU detectable. In table 3 the latter are listed according to the different insertion sites. Of these patients, 18 showed additional signs of local inflammation, indicating local infection.

**Table 2:** Bacterial contamination after removal of the catheter (total number of catheters: n = 198). CFU = colony forming units; the presence of less than 15 CFU of a single organism per catheter is considered to indicate catheter contamination, higher counts are defined as catheter colonization.

No bacterial catheter contamination	n = 151 (76.3%)		
Bacterial catheter contamination	n = 47 (23.7%)		
One organism per catheter	n = 31 (66% of contaminated catheters)		
Two or more organisms per catheter	n = 16 (34% of contaminated catheters)		
Total organisms	n = 66 (= 100 %)	≤15 CFU	≥15 CFU
Normal skin flora			
Coagulase negative staphylococci	n = 40 (60.6%)	10	30
Bacillus species	n = 9 (13.6%)	5	4
Enterococcus species	n = 3 (4.5%)	2	1
Optional pathogenic			
Escherichia coli	n = 5 (7.8%)	5	0
Enterobacter species	n = 3 (4.5%)	2	1
Klebsiella species	n = 3 (4.5%)	3	0
Morganella morganii	n = 1 (1.5%)	1	0
Nonfermenter species	n = 1 (1.5%)	1	0
Pseudomonas aeruginosa	n = 1 (1.5%)	0	1

**Table 3:** Catheter colonization rate at the different sites of insertion. The presence of more than 15 CFU (colony forming units) of a single organism per catheter is defined as catheter colonization, and more than 15 CFU accompanied by local signs of inflammation indicate local infection. Values are expressed as median (25. / 75. percentile) or absolute and relative incidences; n = (%).

Localization	Total number of catheters	Duration of catheter use (days)	Not colonized n (%)	Colonized n (%)	Colonized *and *local infectious signs; n (%)
Epidural catheter (C6/7 - T2/3)	29	6 (5 / 8)	29 (100%)	0 (0%)	0 (0%)
Epidural catheter (T3/4 - T 12/L1)	59	4 (3 / 6)	51 (86.4%)	8 (13.6%)	4 (6.8%)
Epidural catheter (L1/2 - L4/5)	8	4 (3 / 6)	7 (87.5%)	1 (12.5%)	0 (0%)
Paravertebral catheter (level T3 - T8)	4	1 (1 / 3)	4 (100%)	0 (0%)	0 (0%)
Psoas compartment	5	4 (3 / 4)	4 (80%)	1 (20%)	1 (20%)
Posterior sciatic (gluteal)	1	2	1 (100%)	0 (0%)	0 (0%)
Distal sciatic (popliteal)	1	2	1 (100%)	0 (0%)	0 (0%)
Anterior sciatic (proximal)	7	2 (1 / 3)	4 (57.1%)	3 (42.9%)	1 (14.3%)
Femoral nerve	68	3 (1 / 3)	50 (73.5%)	18 (26.5%)	10 (14.7%)
Interscalene plexus	9	2 (1 / 3)	8 (88.9%)	1 (11.1%)	1 (11.1%)
Infraclavicular plexus	5	3 (2 / 6)	4 (80%)	1 (20%)	1 (20%)
Axillary plexus	2	3 (1 / 4)	2 (100%)	0 (0%)	0 (0%)
Total	198	3 (2 / 5)	151 (83.3%)	33 (16.7%)	18 (9.1%)

### Results of the logistic regression analysis

The stepwise logistic regression analysis revealed that out of the 26 potentially relevant parameters only three independent factors remained in the final model as statistically significant (table 4). Catheter placement in the groin was associated with a significant higher incidence of catheter colonization (p = 0.004). The odds-ratio was 3.4 (95%-confidence interval: 1.5 – 7.8) compared to all other anatomical sites. No other potential risk factor that can be determined preoperatively came out as statistically significant. Postoperatively, removal of the catheter dressing, either intentionally or unintentionally, was associated with an increased risk for colonisation. Using the graphical exploratory tools in the JMP 5.1 software, there was an almost linear increase of the rate of colonization with an increasing number of changes of the dressings. Results of the logistic regression analysis revealed that each attempt to change the dressing increased the risk with an odds ratio of 2.1 (95%-CI: 1.4 – 3.3; p = 0.001). There was a maximum number of changing the dressing of five times.

**Table 4:** Results of the final step of the backward logistic regression analysis. Three factors remained in the final model and are presented with their odds ratio and 95%-confidence interval. The coefficient of each factor can be used with the constante of the model to calculate a predicted risk for each patient

	Coefficient	Standard error	P	Odds ratio (95%-confidence interval)
Catheter placement in the groin	1.222	0.424	0.004	3.39 (1.48 – 7.79)
Changing the dressing (per attempt)	0.753	0.222	0.001	2.12 (1.37 – 3.29)
Postoperative administration of an antibiotic (at least for 24 hours)	- 0.896	0.516	0.05	0.41 (0.12 – 1.02)
Intercept	- 2.634	0.414	< 0.0001	

Postoperative administration of an antibiotic drug at least for 24 hours significantly reduced the risk of catheter colonization. The odds-ratio was 0.41 (95%-CI: 0.12 – 1.0; p = 0.05). The constant of the equation of the regression analysis (- 2.63) and the coefficients for each risk factor can be used to calculate a predicted risk for each patient. This theoretical risk can vary between 2.8% (when a catheter is not placed in the groin, no changing of the dressing is performed, and the patients receives postoperative antibiotic treatment) and 91% (in a patients receiving a femoral nerve catheter, with no postoperative antibiotic treatment, and where the dressing was removed five times or more). These calculations are performed for demonstration in the appendix. The goodness of fit was moderate but acceptable (Nagelkerke's R^2 ^= 0.20).

### Clinically infected catheters

Despite the high rates of catheter colonization and superficial local infection, only two clinical infections occurred.

On the fourth postoperative day (the dressing was changed once on the second postoperative day) a patient with an interscalene plexus catheter developed pain at the insertion site, neuropathic pain of the arm and a reddish swelling of 4 cm in diameter, temperature of 38.6°Celsius, and a leukocyte count of 16.7 G·l^-1 ^within a few hours. Until then, the patient did not receive any prophylactic antibiotic except for the intraoperative single-shot administration of 1.5 g intravenous cefuroxim. The catheter was immediately removed and antibiotic therapy with cefuroxim 1.5 g intravenously three times daily was initiated. All symptoms disappeared within the following two days. Two different species of coagulase negative staphylococci (staphylococcus epidermidis) were found on the catheter tip, both of them with CFU > 15. One kind of staphylococcus epidermidis was resistant to cefuroxim, but since the symptoms resolved quickly, the antibiotic regimen was not changed.

The other patient presenting with an infectious complication had an epidural catheter at T7/8. The dressing was changed three times. Only the perioperative single-shot antibiotic with a fix combination of 2 g ampicillin + 1 g sulbactam had been administered, and no further antibiotic treatment was necessary. On the fifth postoperative night he developed very intensive pain and a dark red swelling of 8 cm in diameter superficially just underneath the skin. Until then, a continuous infusion of 4–6 ml/h ropivacaine 0.2% was infused. Neurological examination was normal, neither were there signs of systemic reaction like fever or leukocytosis. The catheter was removed and a local disinfectant ointment was applied. Within 36 hours all symptoms had resolved. The bacterium found on the catheter tip was again staphylococcus epidermidis with > 15 CFU.

## Discussion

In this study, catheter colonization occurred with an incidence of almost 17%. More than half of these colonized catheters also presented with local signs of inflammation (9%). In contrast to these high colonization rates real catheter related infections (local complications, bacteriaemia and / or systemic reactions like fever and leukocytosis) are quite rare. Cuvillon found only three out of 208 femoral catheters with transitory bacteriemia likely related to the catheter, and no abscess occurred, despite the high colonization rate of 57% [14]. Steffen et al. reported a low incidence of colonization in a series of 502 epidural catheters. Several large studies reported epidural abscesses with a varying incidence between 0% and 3% [5,15-17].

In our trial only two catheter related local infections occurred. Both resolved completely within two days with only local ointment or intravenous antibiotics. No serious complication occurred at all during our observational period.

Using a multifactorial statistical model, three independent factors could be identified that were associated with bacterial colonization. However, only one factor (anatomical localization of the insertion site) can be used as a "true risk factor" since the other risk factors are "postoperative" variables. E.g., the decision to perform antibiotic therapy is often performed by the surgeon and the number of changing of the dressing is not easy to foresee.

All other potential "true risk factors" that are patient related factors (e.g. gender, age, pre-existing diseases), puncture site and technical details of catheter placement and fixation (e.g. number of attempts until successful placement, catheter tunnelling) were removed as insignificant. This means that it is not possible to discriminate which patient will or will not develop catheter colonization preoperatively. This result highlights the need for a close postoperative evaluation of every patient even if no factor is present that has been described as a risk factor in previous reports.

Age, preexisting diseases or medical treatment which compromise the immune response have been discussed as potential risk factors and in part are proven risk factors for surgical wound infection [18]. In our trial, neither age nor preexisting diabetes mellitus, cancer disease, infectious disease, abscess in the past, infection with other catheter material in the past, prolonged corticosteroid therapy or short term corticosteroid therapy perioperatively were indicators for an increased risk. Furthermore, combining disease states that occurred too infrequent to have a realistic change to achieve statistical significance did also not lead to variables with significant impact.

The site of catheter insertion is another potential influencing factor in previous studies. The femoral site was associated with a rate of bacterial colonization as high as 57% [14], whereas the popliteal insertion site had a very small bacterial colonization rate of 7.5% [19]. Epidural catheters revealed catheter colonization in 6 to 35% [2,20]. One possible explanation for these differing results might be the great variations with respect to the density of sebaceous glands in the different insertion sites that has been shown to impact the ability of local disinfectants to reduce the number of microorganisms [21]. For example Steffen et al. [2] reported a higher incidence of colonized catheters in patients where the epidural catheters were placed at a thoracic level compared to the lumbar route. However, a variable that should distinguish between potentially more contaminated and clean puncture sites based on the latter hypothesis was early removed as insignificant in our analysis. Catheter placement in the groin (femoral nerve catheters and sciatic catheters advanced via the anterior approach) was associated with a significantly higher incidence of colonization than all other anatomical landmarks.

Technically difficult catheter insertion may cause asymptomatic haematoma that may later become the focus of bacterial colonization [6]. However, this theory was not supported by other authors [5]. In our trial the numbers of skin perforations with the needle during catheter placement did not increase the occurrence of catheter colonization.

The repetitive administration of antibiotics during the postoperative period reduced the incidence of catheter colonization. Reports from the literature support the view that antibiotic therapy during the perioperative period lowers the risk for infectious catheter complications. A relatively high rate of epidural abscess occurred in a population that apparently did not receive perioperative antibiotics routinely [5]. Furthermore, in a series of 405 axillary catheters, the only abscess occurred in a patient who had not received an antibiotic [22]. It is interesting to notice that intraoperative single dose antibiotic treatment did not provide sufficient protection. However, this single shot treatment was usually administered 30–60 minutes after the insertion of the epidural or peripheral nerve catheter. Thus, we can not answer the question, whether antibiotic prophylaxis *before *catheter placement might be able to reduce the incidence of colonization.

Concerning the possible routes for catheter colonization, Hunt et al. demonstrated that the catheter hub represented the main route for catheter colonization [11]. Therefore disconnection of the closed system or filter changing maneuvers should be avoided if possible [11,23]. We analyzed the situations where catheters were accidentally disconnected assuming that the unprotected end was open for an indefinite time and could let microorganisms pass through. In the multifactorial analysis, accidental disconnection of the catheter was removed at a late stage of the stepwise logistic regression procedure. Thus, this potential risk factor was insignificant but is a candidate for further investigations.

Local anaesthetic solutions with bacteriostatic effect like bupivacaine, prilocaine, lidocaine and tetracaine [24] have shown to decrease the risk of infection [8,9]. In our trial, only ropivacaine 0.2% was used postoperatively for continuous administration and thus this potential influencing factor could not be included in the statistical model.

Duration of catheter use has been found to increase the risk of infectious complications in a Danish study with epidural catheters [5]. No epidural abscess was found with use of catheters ≤ 2 days, but one third of the abscesses were found in patients who had the catheter in situ for three days only. This implicates that even a short catheterization time of three days does not eliminate the risk of infection. In another observational trial, there was a very strong correlation between duration of catheter use and infectious complications in patients with perfusion disorders, but not in the other subgroups [25,26]. In our own trial we could not observe a statistically significant time dependency, but the variable was late removed at step 21 of 23. In our trial catheters were removed between day 0 and day 31. The decision to withdraw a catheter was primarily based on the daily pain evaluation by the patient. However, also local signs of the insertion site influenced the decision to remove the catheter. Thus, it is important to notice that duration of catheter use is not a risk factor only under the strict assumption that the site of insertion is evaluated at least once a day and the catheter is immediately removed if there are any signs of local redness, swelling or pain at the insertion site. This is in agreement with a recent study showing that the duration of use of an epidural catheter was not different in colonization and in sterile catheters [2]. Attention should be paid to the fact that duration of catheter placement has some correlation with the number of removal of catheter dressing (Pearson correlation coefficient r = 0.50; Spearman correlation coefficient rho = 0.35). Only the latter factor remained statistical significant in the final model, and thus some of the predictive information provided by the duration of catheter placement was virtually transferred. This phenomenon of co-linearity is discussed in more detail in the following paragraph.

### Limitations of the study

Several of the potential risk factors that were evaluated using a stepwise logistic regression analysis are correlated with each other. For example there is a strong correlation between all factors describing the pre-anaesthetic health condition of a patient summarized as the ASA-status on one hand, and cancer disease or age on the other hand. Thus, if one or several of such factors are removed during the stepwise exclusion procedure a certain part of the information of the removed factor is transferred to the correlated factors still in the model. This can lead to artifacts by increasing the reputed impact of a risk factor. Thus, it is important to notice that the risk factors that remained in the final model are not necessarily causal for the rate of catheter colonization but are maybe only associated with an increased incidence. Example given: it could be misleading to reason that the underlying reason why the number of changing the dressing increases the risk for catheter colonization is simply the manipulation of the catheter (with subsequent contamination etc.). Rather it is also possible that other correlated factors (contamination with blood, local secretion at the insertion site of the catheter, intensive sweating of the patient, and the site of catheter placement) are the "true" (causal) factors for colonization and were erroneously removed during the logistic regression procedure transferring their predictive power to other correlated factors.

Another limitation of the study is the lack of a sufficient number of all catheter techniques at all anatomical sites. E.g., there were only two axillary plexus catheters and from a theoretical point of view this anatomical site has similar problems as the groin with respect to difficulties in fixation of the catheter, intense sweating, a high rate of sebaceous glands etc. Thus, the fact that the axilla and other anatomical sites underrepresented in this study were not identified as a relevant risk factor, does not necessarily mean that these locations are not associated with a higher incidence of catheter colonization.

## Conclusion

Summarizing the present results, three independent risk factors could be detected applying a stepwise logistic regression procedure to a great number of potential risk factors for bacterial catheter colonization. Catheter localisation in the groin, removal of the dressing and omission of postoperative antibiotics were associated but not necessarily causal for postoperative catheter colonization.

## Appendix

Calculation of the theoretical risk for bacterial colonization of a peripheral or epidural nerve catheter, defined as more than 15 colony forming units.

Patients 1 (low risk) received an epidural catheter for five days after hemicolectomy. During this time, removal of the dressing was not necessary. He received an antibiotic prophylaxis during the first three postoperative days.

The risk for colonization of the epidural catheter can be calculated as follows:

- z = - (-2.63_constante _+ (-0.90 × 1_antibiotic treatment _+ 0.75 × 0_number of dressing replacement _+ 1.22 × 0_localisation: not groin_) = - 3.53 Risk [%] = 100% / (1+e^-z^) = 100% / (1+e^-3.53^) = 2.8%

Patient 2 (high risk) received a sciatic nerve catheter for five days using the anterior approach (in the groin). Postoperatively, the dressing had to be changed every day. The patient received only a single dose of an antibiotic intraoperatively.

- z = - (-2.63_constante _+ (-0.90 × 0_antibiotic treatment _+ 0.75 × 5_number of dressing replacement _+ 1.22 × 1_localisation: groin_) = - (-2.34) Risk [%] = 100% / (1+e^-z^) = 100% / (1+e 2.34) = 91.2%

## Competing interests

The author(s) declare that they have no competing interests.

## Authors' contributions

AMM designed the study, collected the clinicaldata and wrote the manuscript.

KMK collected the clinical data.

MK performed the culture techniques.

RN collected the clinical data.

VER collected the clinical data.

HW participated in the conception of the study.

SZ performed the culture techniques.

LHJE designed the study, performed the statistical analysis and extensively revised the manuscript.

All authors read and approved the final manuscript.

## Pre-publication history

The pre-publication history for this paper can be accessed here:


